# Dynamics of *Yersinia pestis* and Its Antibody Response in Great Gerbils (*Rhombomys opimus*) by Subcutaneous Infection

**DOI:** 10.1371/journal.pone.0046820

**Published:** 2012-10-05

**Authors:** Yujiang Zhang, Xiang Dai, Xinhui Wang, Abulimiti Maituohuti, Yujun Cui, Azhati Rehemu, Qiguo Wang, Weiwei Meng, Tao Luo, Rong Guo, Bing Li, Abulikemu Abudurexiti, Yajun Song, Ruifu Yang, Hanli Cao

**Affiliations:** 1 The Center for Disease Control and Prevention of the Xinjiang Uygur Autonomous Region, Urumqi, China; 2 Laboratory of Analytical Microbiology, State Key Laboratory of Pathogen and Biosecurity, Beijing Institute of Microbiology and Epidemiology, Beijing, China; University of Helsinki, Finland

## Abstract

**Background:**

*Rhombomys opimus* (great gerbil) is a reservoir of *Yersinia pestis* in the natural plague foci of Central Asia. Great gerbils are highly resistant to *Y. pestis* infection. The coevolution of great gerbils and *Y. pestis* is believed to play an important role in the plague epidemics in Central Asia plague foci. However, the dynamics of *Y. pestis* infection and the corresponding antibody response in great gerbils have not been evaluated. In this report, animal experiments were employed to investigate the bacterial load in both the liver and spleen of infected great gerbils. The dynamics of the antibody response to the F1 capsule antigen of *Y. pestis* was also determined.

**Methodology:**

Captured great gerbils that tested negative for both anti-F1 antibodies and bacterial isolation were infected subcutaneously with different doses (10^5^ to 10^11^ CFU) of a *Y. pestis* strain isolated from a live great gerbil during routine plague surveillance in the Junggar Basin, Xinjiang, China. The clinical manifestations, changes in body weight, anal temperature, and gross anatomy of the infected animals were observed. The blood cell count, bacterial load, and anti-F1 antibody titers were determined at different time points after infection using a blood analyzer, plate counts, and an indirect hemagglutination assay, respectively.

**Conclusions/Significance:**

The dynamics of bacterial load and the anti-F1 antibody concentration in great gerbils are highly variable among individuals. The *Y. pestis* infection in great gerbils could persist as long as 15 days. They act as an appropriate reservoir for plague in the Junggar Basin, which is part of the natural plague foci in Central Asia. The dynamics of the *Y. pestis* susceptibility of great gerbil will improve the understanding of its variable resistance, which would facilitate the development of more effective countermeasures for controlling plague epidemics in this focus.

## Introduction

*Rhombomys opimus* (great gerbil) is widely distributed in the barren and semi-barren desert areas of Central Asia, including Northwestern China, the Mongolian Republic, Russia, Kazakhstan, Iran, and Afghanistan [1], [2]. The great gerbil is a social rodent with a family-habitat lifestyle. A family of great gerbils is generally composed of one male, two to six females, and several offspring [3]. Its burrow structure is very complex, consisting of several hundreds to thousands of entry openings and tunnels up to 100 meters in length, with all tunnels and entrances connected with each other [4]. The burrows of great gerbils are distributed in island-like patterns, which greatly contribute to the ecological system in the barren desert areas of Central Asia. *Yersinia pestis* has been isolated from this rodent and its parasitic fleas, which indicates that the great gerbil is a major reservoir that maintains natural plague foci [5]. Davis et al. suggested that the family-oriented lifestyle of great gerbils is an essential component for plague epidemics [6]. The complex population structure of the species, the abundance of parasitic fleas, and large variations in the susceptibility of great gerbils to *Y. pestis* infections leads to variable and complex plague epidemics among the rodents in a given area [7]. Based on their long-term observations of the plague epidemics in great gerbils in Kazakhstan, Davis et al. proposed that the weather, the population structure of the reservoir, along with its dynamics, the invasion and spread of the reservoir, the *Y. pestis*-resistance of the reservoir, and population structure of the flea species that parasitizes the reservoir, and their dynamics are the key factors that determine plague epidemics in rodents in this focus [4], [6]. Their group also indicated that the coevolution of great gerbils and *Y. pestis* plays an important role in these plague epidemics [6], [8].

Long-term surveillance of plague epidemics in great gerbils demonstrated that although a large number of *Y. pestis* strains can be isolated from both the rodents and their fleas during severe plague epidemics, dead rodents for bacterial isolation are difficult to find during non-epidemic periods [5], [7]–[9]. This phenomenon has not been observed in other natural plague foci in both China and other countries [10]–[13], such as in the Qinghai–Tibet marmot (*Marmota himalayana*) plague focus, where *Y. pestis* could be isolated from 69.57% of the dead marmots found in the wild during non-epidemic periods. In other natural plague foci, plague pathogens could also be isolated from dead *Citellus undulatus*, *Meriones unguiculatus*, and *Microtus fuscus*. Surveillance of antibodies against the F1 capsule antigen in great gerbils show that the average antibody positive rate is approximately 10% in the Junggar Basin of China, with some areas reaching as high as 30% to 70% [9]. This rate can be partially explained by the high resistance of great gerbils to *Y. pestis* infections. The susceptibility dynamics of the great gerbil to *Y. pestis* infection was therefore investigated to improve the current understanding of the variable resistance of this rodent species and to improve the effectiveness of countermeasures for controlling plague epidemics in this area.

## Materials and Methods

### Bacteria and Animals

The *Y. pestis* strain 2505 was isolated by our laboratory from a live great gerbil in 2005 during routine plague surveillance in the Junggar Basin. This strain is negative for nitrate reduction and rhamnose fermentation, but is positive for arabinose fermentation, with a median lethal dose (LD_50_) of 10 CFU (colony forming units) for mice and 1,660 CFU for guinea pigs [14]. Great gerbils were captured as experimental animals from the natural plague focus in the Junggar Basin. An indirect hemagglutination assay (IHA) and a reverse IHA (RIHA) [2] were employed to detect anti-F1 antibodies and F1 antigens in the captured animals, respectively. The animals that were negative for anti-F1 antibodies and F1 antigens were reared in the laboratory for six months. Before the animals were challenged with *Y. pestis*, the anti-F1 antibodies were assayed at least twice to exclude the antibody-positive animals. Finally, 40 adult great gerbils (>110 g in body weight) and 30 juvenile great gerbils (<100 g in body weight) were used for the *Y. pestis* susceptibility assay. Furthermore, 90 gerbils with an average body weight of 129.0 g±11.1 g were used to observe the *Y. pestis* dynamics in both the liver and spleen of the great gerbils. Finally, 40 animals with an average body weight of 145.5 g±9.0 g were employed to study the dynamics of the F1 antibody response in the serum of great gerbils. Guinea pigs were purchased from the Xijiang Experimental Animal Center for gross anatomy comparisons. The full details of this study were approved by the ethics committee of the Center for Disease Control and Prevention of the Xinjiang Uygur Autonomous Region in China.

### Susceptibility of Great Gerbils to *Y. pestis*

The animal challenge experiments were performed according to the biosafety regulations issued by the Ministry of Health, China. The 40 adult great gerbils were divided into 8 groups, with 5 animals in each group (Table 1). One group was used as the control, with a subcutaneous injection of an equal amount (1 ml) of physiologic saline to the groin, whereas the other 7 groups were challenged with varying doses of *Y. pestis* strain 2505 from 7.4×10^5^ CFU to 7.4×10^10^ CFU, and 3×10^11^ CFU. The 30 juvenile animals were divided into six groups, with five animals in each group (Table 1). One group served as the control, whereas the other five were used as experimental groups with varying challenge doses ranging from 7.4×10^6^ CFU to 17.4×0^10^ CFU.

**Table 1:** Susceptibility of adult and juvenile great gerbils to subcutaneous challenge by *Y. pestis*.

Age	Group	Number (♀, ♂)	Weight, mean ± SD (g)	Challenge dose (CFU)	Died/total exposed	No. of deaths*	Days of death after challenge	Positive no. of F1-Ab/bacterial isolates$	Survival rate (%)
Adult	1	5(4,1)	139.9±8.0	7.4×10^5^	1/5ζ	0		1/0	80
	2	5(4,1)	140.2±7.3	7.4×10^6^	1/5ζ	0		4/0	100
	3	5(4,1)	141.1±6.9	7.4×10^7^	1/5ζ	0		4/0	100
	4	5(4,1)	141.9±6.6	7.4×10^8^	2/5ζ	1	3	3/1	60
	5	5(4,1)	142.7±6.9	7.4×10^9^	0/5	0		5/0	100
	6	5(4,1)	143.9±6.6	7.4×10^10^	4/5	4	3, 4, 4, 4	5/4	20
	7	5(3,2)	142.9±6.1	3.0×10^11^	2/5ζ	1	7	4/1	60
	control	5(3,2)	153.7±4.5	0	0/5	0		NAψ	100
Juvenile	8	5(3,2)	77.8±5.3	7.4×10^6^	2/5ζ	1	5	3/1	60
	9	5(3,2)	77.6±4.5	7.4×10^7^	0/5	0		5/0	100
	10	5(2,3)	78.2±5.1	7.4×10^8^	1/5ζ	0		4/0	80
	11	5(2,3)	78.4±4.9	7.4×10^9^	0/5	0		5/0	100
	12	5(3,2)	78.5±5.1	7.4×10^10^	2/5ζ	1	3	4/1	60
	Control	5(3,2)	79.2±5.3	0	0/5	0		NA	100

* :The dead animals were considered to die specifically from *Y. pestis* infection because *Y. pestis* was isolated from both their liver and spleen.

ζ :One animal died non-specifically.

$ :Live animals, from which *Y. pestis* were isolated or antibodies were detected on day 21 p.i., were considered infected specifically with *Y. pestis*.

ψ :NA stands for “not applicable”.

**Figure 1:**
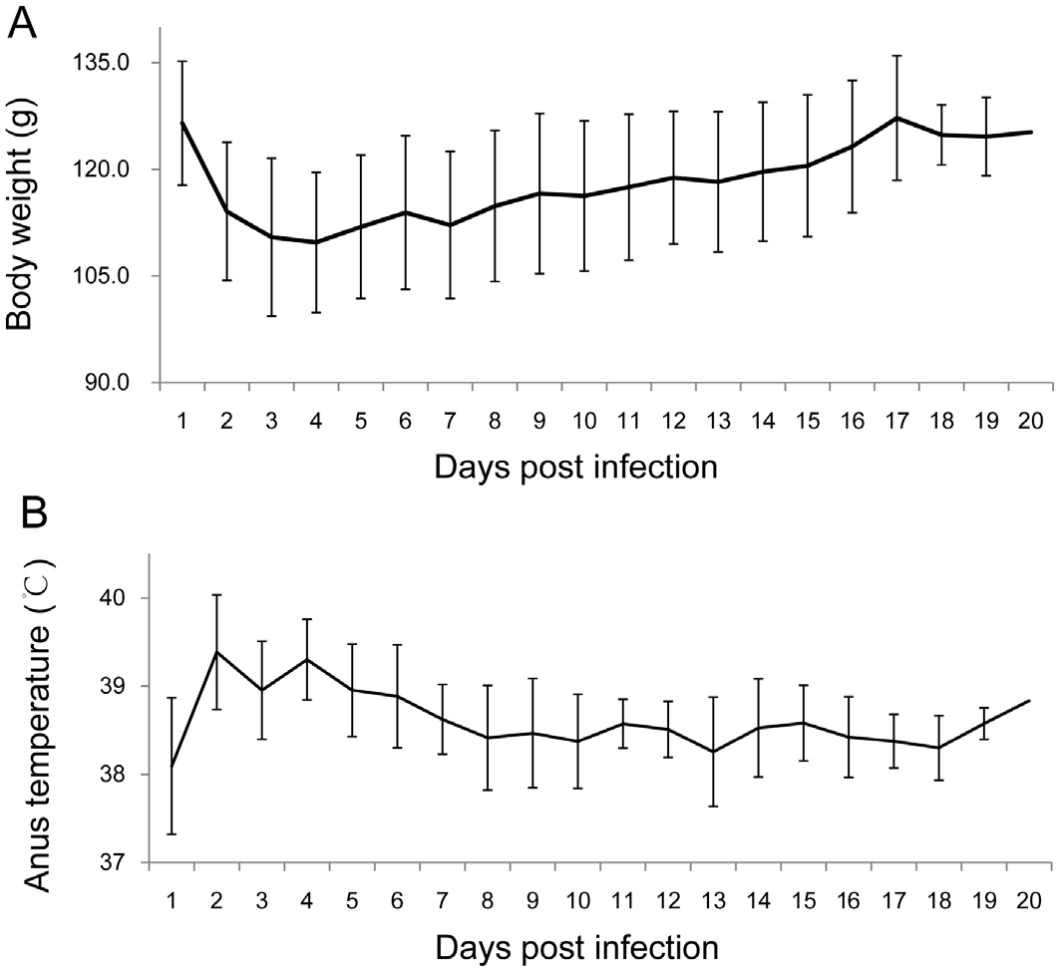
Post infection changes in the average body weight (A) and average anal temperature (B). Approximately 2.0×10^9^ CFU of *Y. pestis* strain 2505 was injected subcutaneously into the groin of 18 great gerbils on day 0, and then observed for changes in body weight and anal temperature. The average body weight and anal temperature of the 18 animals were employed to represent the changing trends at different time points (days) post infection.

The anal temperature, body posture, and body weight of the animals were observed daily, as well as changes in their fur, respiration, and diet.

*Y. pestis* was isolated using Luria–Bertani (LB) plates at 28°C from the spleen and liver of the dead animals. The cell cultures were confirmed using a phage lysis assay [15]. Sera were also collected for anti-F1 antibody determination. The dead animals that were infected with *Y. pestis* or that tested positive for the F1 capsule antigen or anti-F1 antibodies were considered to have died from *Y. pestis* infection.

The experimental animals were observed for 30 days after the *Y.* *pestis* challenge. After the observation period, all surviving animals were euthanized and necropsied to check for gross anatomic changes and to isolate the bacterium.

**Figure 2:**
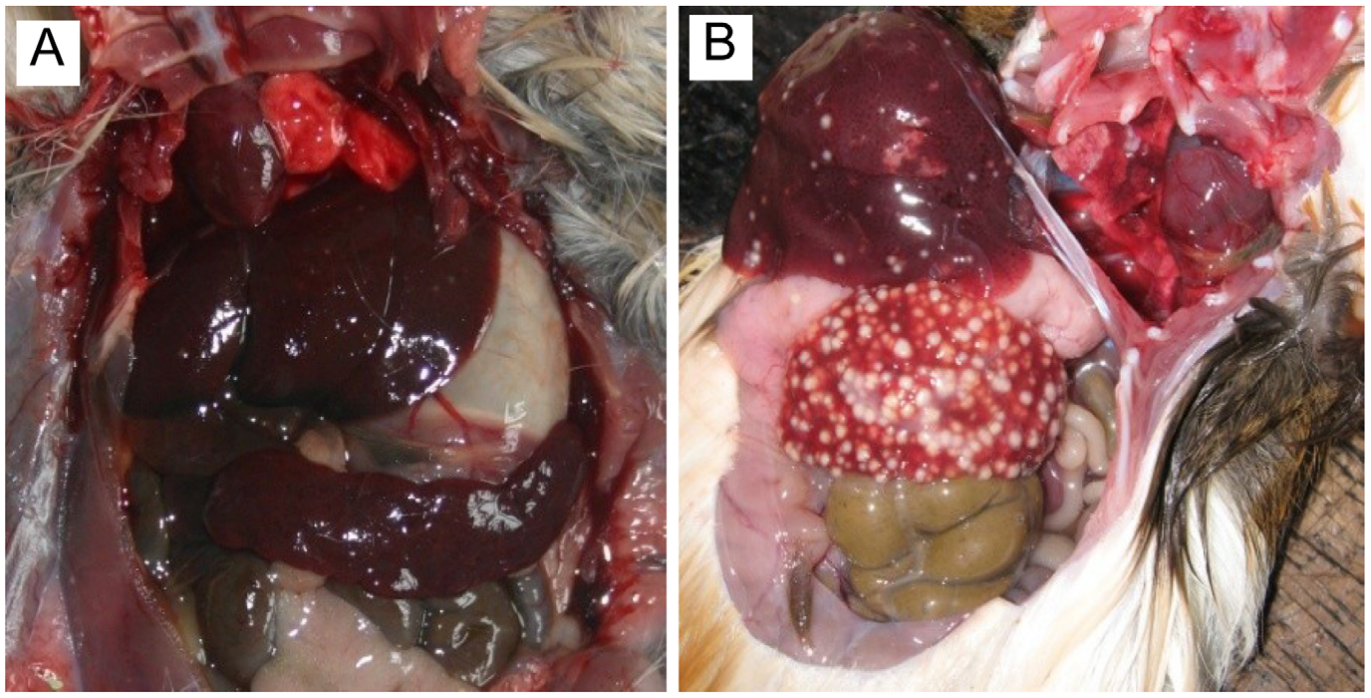
Gross anatomic changes in the liver and spleen of great gerbils (A) and guinea pigs (B). Approximately 7.4×10^10^ CFU of *Y.* *pestis* strain 2505 was injected subcutaneously into the groin of great gerbils, whereas around 5.0×10^4^ CFU was injected subcutaneously into the groin of guinea pigs. The animals were dissected immediately after death on day 3 p.i. The abscesses are clearly seen on the surface of both the liver and spleen of guinea pigs, but no abscesses were observed on the corresponding organs of great gerbils.

### Dynamics of *Y. pestis* in the Different Organs of Great Gerbils

A total of 90 anti-F1 antibody–negative great gerbils (53 female and 37 male) were employed for the bacterial dynamics study. Six were assigned as the control group, which were injected with 1 ml of physiologic saline. The other 84 were challenged with a subcutaneous injection of 2.0×10^10^ CFU of *Y. pestis* into the groin. The animals were observed for 21 days post infection (p.i.). A few of the animals were euthanized daily to collect blood to measure blood parameters on a blood analyzer (MaiRui BC-2800Vet, China), including the white blood cell count, red blood cell count, platelet count, hemoglobin content, mean corpuscular volume, lymphocyte count, monocyte count, and granulocyte count, and to detect the F1 antigen. The livers and spleens were also collected to isolate *Y. pestis*. From days 1 to 7 p.i., five to six animals were euthanized daily; from days 8 to 15 p.i., four animals were sacrificed daily; and from days 16 to 21 p.i., two animals were sacrificed daily.

One tube of anticoagulated blood was used for blood parameter analysis and serum was collected from the coagulated blood to determine the F1 antibody concentrations via IHA [2]. The liver and spleen samples were first weighed, and then homogenized by adding 10 ml of physiologic saline to the intact liver and 2 ml to the spleen. Then, 0.1 ml of the supernate was spread on triplicate LB agar plates and incubated at 28°C for 24 h to 48 h. The mean CFU was recorded for each sample. Meanwhile, the F1 antigen was also detected by RIHA, as mentioned above.

**Table 2:** Dynamics of bacterial load (BL) in both the liver and spleen of great gerbils challenged with 2.0×10^10^ CFU of *Y. pestis*.

Days p.i.	No. of animals sampled	No. of survivors^ζ^	No. of Ab positive	No. ofinfected	No. of specific death	Survival (%)	Bacterialisolationrate (%)	BL in liver (CFU/g)		BL in spleen (CFU/g)	
								Dead	Survived	Dead	Survived
1	6	4	0	1	0	66.7	16.7	0	0	0	3.46×10^2^
2	5	3	0	4	2	60.0	80.0	4.15×10^4^	1.50×10^4^	3.47×10^7^	8.06×10^7^
3	6	2^ζ^	0	4	3	33.3	66.7	3.01×10^5^	1.73×10^4^	7.75×10^9^	3.89×10^3^
4	5	2^ζ^	0	4	2	40.0	80.0	3.44×10^10^	7.95×10^3^	1.23×10^11^	6.76×10^5^
5	6	4	1	5	2	66.7	83.3	4.18×10^7^	1.50×10^4^	1.98×10^10^	0
6	6	4^ζ^	0	4	1	66.7	66.7	3.44×10^5^	1.68×10^4^	8.06×10^7^	1.10×10^3^
7	5	5	2	4	0	100.0	60.0		1.85×10^4^	8.68×10^7^	0
8–21	45	45	38	38	0	100.0	4.4		4.31×10^1^ *		1.76×10^3^ **
Total	84	69	41	64	10	82.1	32.1				

* : At day 14 p.i., *Y. pestis* was isolated from the liver of a live animal with a bacterial load of 4.31 CFU/g.

** : At day 15 p.i., *Y. pestis* was isolated from the spleen of a live animal with a bacterial load of 176 CFU/g.

ζ: Six great gerbils died of non-specific causes.

### Determination of *Y. pestis* F1 Antibody Response Dynamics under Different Challenge Dose

Up to 40 great gerbils (28 female and 12 male) negative for anti-F1 antibodies were divided into seven experimental groups and one control group, with 5 animals in each group. The experimental groups were injected with 7.4×10^5^ CFU to 7.4×10^10^ CFU of *Y. pestis* in 1 ml of physiologic saline into the groin. For the control group, 1 ml of physiologic saline was injected.

Blood samples were collected on days 5, 7, 15, 30, 60, 90, and 120 p.i. from the tail of the great gerbils to detect anti-F1 antibodies by IHA.

**Table 3:** Dynamics of the F1-antibody in great gerbils challenged by different doses of *Y. pestis.*

Challenge dose (CFU)	No. of samples	No. of positive	Antibody titers (mean ± SD)/No. of animals positive for Ab					
			5 d	7 d	15 d	30 d	60 d	120 d
7.4×10^10^	3	3	(0.33±0.58)^a^/1	(2.33±0.58)/3	(4.33±1.15)/3	(6.0±1.0)/3	(7.33±1.15)/3	(8.33±1.53)/3
7.4×10^9^	4	4	0	(0.5±0.58)/2	(2.5±0.58)/4	(3.25±1.50)/4	(3.75±0.96)/4	(4.33±1.53)/3^a^
7.4×10^8^	4	3	0	(0.25±0.50)/1^b^	(1.75±1.26)/3	(2.50±1.73)/3	(4.33±0.58)/3	(4.0)/1^c^
7.4×10^7^	5	4	0	0	(2.0±1.22)/4	(3.0±1.73)/4	(4.25±0.5)/4	(5.33±0.58)/3^a^
7.4×10^6^	4	3	0	0	(1.75±1.09)/3	(3.0±2.24)/3	(4.0±0.82)/3	(5.5±0.5)/2^a^
7.4×10^5^	4	1	0	0	0	(1.0±2.0)/1^b^	(4.0±0.0)/1	–b

a :The antibody titer was recorded as the dilution time, by setting 1∶8 as “1”, 1∶16 as “2”, 1∶32 as “3”, and 1∶4096 as “10”. The antibody was determined by IHA and each sample was repeated three times.

b :Only one of the 4 animals produced F1-Ab and there was no measurable F1-Ab for the other three animals.

c :Only one animal was available for F1-Ab determination because of the unexpected death of the other three animals.

## Results

### Clinical Manifestations of the Great Gerbils by *Y. pestis* Infection

The clinical manifestations of *Y. pestis* infection in the great gerbils varied greatly. The animals generally showed no symptoms at the lower challenge doses (<7.4×10^7^ CFU). At higher challenge doses (>7.4×10^8^ CFU), the great gerbils manifested signs of infection, including polydipsia, closed eyes, poorly groomed hair, and hunched posture, as well as reluctance and difficulty in moving. The group challenged with 7.4×10^9^ CFU also showed no signs of infection.

From Table 1, no significant differences were observed between the adult group and juvenile group in terms of susceptibility to subcutaneous challenge with *Y. pestis*. Furthermore, the great gerbils showed highly variable susceptibility to *Y. pestis* infection, which makes this species an ideal reservoir for maintaining the plague focus in the Junggar Basin.

The changes in anal temperature and body weight of the challenged animals were also determined (Figure 1). The anal temperature increased rapidly within the first three days p.i. from 38.1°C±0.8°C before challenge to 39.2°C±0.4°C (*t*-test, *P*<0.0001) p.i., and then gradually dropped to normal 38.1°C±0.76°C by day 7 p.i. The body weight (110.8 g±9.7 g) on day 3 p.i. was lower by 11% compared with that (123.6 g±9.3 g) before challenge (*t*-test, *P*<0.0001), then their body weight gradually increased to that of the normal control (126.5 g±8.7 g) by day 15 p.i.

Most of the blood parameters remained stable during the 21-day observation period after the *Y. pestis* challenge, except for the white blood cell count. The number of white blood cells was significantly increased in two animals during the early challenge period (29.7×10^9^/L on day 3 and 35.4×10^9^/L on day 4 p.i., respectively, for each animal compared with the 4.05×10^9^/L±0.5×10^9^/L in other infected animals and 4.1×10^9^/L±0.55×10^9^/L in the normal control group). The number of lymphocytes increased the most (92.9%, 27.6×10^9^/29.7×10^9^), whereas the percentage of neutrophils (1.0%, 0.3×10^9^/29.7×10^9^) and monocytes (5.7%, 1.7×10^9^/29.7×10^9^) were very small.

According to the gross anatomical observations, the liver and spleen of the challenged great gerbils did not change significantly, which differed from what was observed in guinea pigs challenged with a lower dosage (Figure 2).

### Dynamics of *Y. pestis* Infection in Great Gerbils

Up to 85 great gerbils were challenged with a subcutaneous injection of 2.0×10^10^ CFU of *Y. pestis* to the groin. The animals were observed for 21 days for survival rate, specific infection rate, specific death rate, and bacterial load in both the liver and spleen (Table 2).

Excluding the six possible non-specific deaths, 79 of the great gerbils were included in the final analysis. Up to 64 gerbils were infected with *Y. pestis*, with an infection rate of 81.0% (64/79), whereas only 10 animals died from *Y. pestis* infection, with a death rate of 12.7% (10/79). These results imply that most of the great gerbils were infected by the high challenge dose, but only a small portion of the infected ones died of the infection. The unique susceptibility of great gerbils to *Y. pestis* infection makes this rodent species an ideal reservoir in the natural plague focus in the barren desert area of the Junggar Basin. The bacteria were isolated from the infected animals only within 1 to 7 days p.i., except for the two animals from which *Y. pestis* was isolated from both the liver and spleen on day 14 and 15 p.i.

On day 1 p.i., *Y. pestis* was detected from the spleen of one out of six live animals (16.7%), with an average bacterial load (BL) of 346 CFU/g. On day 2 p.i., the BLs in both the liver and spleen increased dramatically. The BLs began to decrease in the dead animals on day 4 p.i., whereas the BLs decreased in the infected live animals starting from day 7 p.i., (Table 2). As expected, the mean BLs (the mean of all BL values regardless of the time point when they were obtained) in both the liver (6.9×10^9^ CFU/g) and spleen (2.51×10^10^ CFU/g) of the dead animals were much higher than those of the infected survivors (1.29×10^4^ CFU/g in the liver and 1.02×10^7^ CFU/g in the spleen). In the live infected animals, the BLs in the liver from day 2 to 7 p.i. did not significantly change (1.51×10^4^ CFU/g±0.38×10^4^ CFU/g), whereas the BLs in the spleen changed abruptly, ranging from 0 CFU/g to 8.06×10^7^ CFU/g.

The presence of anti-F1 antibodies was detected in 18 of the 21 great gerbils from day 14 to 21 p.i., with a seroconversion rate of 85.7%, which is similar to that from the bacterial isolation results (81.0%; Table 2).

### Dynamic of Anti-F1 Antibodies in Serum

As shown in Table 3, the antibody response of the great gerbils varied significantly among the different challenge groups. At dose of 7.4×10^5^ CFU, only one animal tested positive for anti-F1 antibodies on day 30 p.i., with a lower titer. At higher doses (>10^8^ CFU), the anti-F1 antibodies were detected on day 5 until 7 p.i. On day 15 p.i., all the experimental animals produced anti-F1 antibodies although the number of animals available for antibody measurement was relatively small. As the challenge dose was increased, the number of animals positive for the anti-F1 antibodies also increased, with higher titers. During the 120-day experimental period, the antibody production increased with the infection time.

## Discussion

The susceptibility of great gerbils to *Y. pestis* infection varies dramatically among individuals [16]. The virulence of the *Y. pestis* isolates from great gerbils from different locations was also diverse, and F1-negative strain has also been isolated from great gerbils from the Bosugen Tract [16]. Several strains have been previously isolated from great gerbils from different locations in the Junggar Basin, but the observed virulence to mice did not differ among these strains by LD50. Prior to the present study, no F1-negative strains had been found. The *Y. pestis* strain 2505 employed in this study is highly lethal to mice and less virulent to guinea pigs [14].

Various theories have been introduced to explain the resistance to plague, such as the isoantigenic blood structure of the host [17], the initial activity of oxygen-sensitive cytocidal systems in neutrophils (which is 2.6- to 8.7-fold higher in great gerbils than in mice) [18] and peritoneal leukocytes [19], the antimicrobial potential of professional phagocytes [20], the formation of *Y. pestis* L. forms in great gerbils [21] that are poorly phagocytosed and persist in phagocytes for a long time [22], and the presence of bacteriophages in the host animal [1]. However, the dynamics of *Y. pestis* infection and the antibody response in great gerbils have not been previously reported. In this study, the dynamics of *Y. pestis* infection were investigated by subcutaneously challenging great gerbils with different doses of *Y. pestis* isolated from a live great gerbil during plague surveillance. The results confirmed that great gerbils are globally resistant (82% survival after infection of 84 animals by 2×10^10^ CFU of *Y. pestis*; Table 2), but has variable susceptibility to *Y. pestis* infection [16]. No pathologic changes were observed after a challenge dose of 8.0×10^4^ CFU and no *Y. pestis* was isolated (data not shown for the preliminary experiment). At a challenge dose of 7.4×10^5^ CFU, only one of five great gerbils was infected, and most of the animals survived from the infection. However, in marmots, a challenge dose of 1.0×10^5^ CFU causes 100% death in experimental animals [23]. The susceptibility of great gerbils to *Y. pestis* infection is much lower than that of *Citellus undulatus*, *Citellus dauricus*, *Rattus flavipectus*, prairie dogs, and cats [12], [24], [25]. Although different *Y. pestis* strains were used for these animal challenge experiments, these results could indicate the different levels of susceptibility of various reservoirs to this pathogen. As the challenge dose increased, the death rate caused by *Y. pestis* infection also increased among the great gerbils, with 80% of the specific deaths caused by a dose of 7.4×10^10^ CFU from day 3 to day 7 p.i. The anti-F1 antibodies were detected as early as day 5 p.i. at this challenge dose.

The clinical symptoms of infected great gerbils were different from those of infected mice, guinea pigs, and cats [24], [25]. The anal temperature increased quickly within the first three days p.i., before it gradually decreased to normal on day 7 p.i. The white blood cell count increased slightly during early infection, which mainly consisted of lymphocytes, and no abscesses were observed on the surfaces of the liver and spleen. These results indicate that the great gerbil is an ideal reservoir for the natural plague foci in the Junggar Basin in Xinjiang, China. No significant differences in *Y. pestis* susceptibility were observed between the adult and juvenile great gerbils (Table 1). Although several reports on the possible mechanisms of the plague resistance of great gerbils have been published, the exact mechanism behind their resistance is still unknown; whole genome sequencing might provide more clues to this unelucidated mechanism [26].

The present results demonstrate that 7.4×10^5^ CFU of *Y. pestis* causes infection in some great gerbils via the subcutaneous route, which was the lowest dose tested in the present study. The required dose to cause an infection might be lower than this dose. However, as mentioned above, a pilot animal experiment with a challenge dose of 8.0×10^4^ CFU was performed but no clinical signs were observed and no *Y. pestis* was isolated from the challenged animals. A dose of 7.4×10^8^ CFU of *Y. pestis* produced a higher death rate in great gerbils. This species is the most resistant rodent to *Y. pestis* among the known reservoirs. At a dose of 2.0×10^10^ CFU, 76.2% of the challenged great gerbils demonstrated symptoms of infection, but only 11.9% (10/84; Table 2) of them died from plague on days 1 to 6 p.i. In terms of the bacterial load in the liver and spleen, significant variations were observed between individuals. The BLs in the dead individuals were much higher than those in the survivors (as high as 5×10^5^ times in the liver and 2×10^3^ times in the spleen). This result suggests that the growth of *Y. pestis* in large quantities within the bodies of the infected great gerbils is an important cause of death. *Y. pestis* was isolated from a few of the infected live individuals on day 14 and 15 p.i., with very low BLs. This result indicates that *Y. pestis* lives in the bodies of great gerbils at low quantities for a long time, keeping a balanced competition between the host and the pathogen. This host–pathogen relationship might be a mechanism of plague silence during non-epidemic periods [27]. An investigation evaluated capability of remaining latent infections of *Y. pestis* in hibernating ground squirrels (*Citellus pygmaeus* Pallas) after the animals were challenged with *Y. pestis* during hibernation [28]. As shown by our experiment, *Y. pestis* could be stay in gerbils for as long as 15 days, which is much longer than other virulent strains of *Y. pestis* from other natural plague foci. Although the example of a hibernating animal is not directly comparable to great gerbils, it provided some clues to the mechanism behind plague silence during non-epidemic periods.

*Y. pestis* was isolated from the spleen and liver of great gerbils on day 1 and day 2 p.i., respectively, which suggests that the pathogen quickly spreads to the spleen and liver immediately after subcutaneous infection, possibly by spreading first to the lymph nodes and then to the organs via the bloodstream [29]. Although the number of animals in each group was limited because of the availability of captured great gerbils, the present results still indicated persistent plague infections in great gerbils.

In the current experiment, more than 80% of the great gerbils were infected at challenge doses exceeding 7.4×10^5^ CFU. This result confirms that the susceptibility of great gerbils to higher doses of *Y. pestis*. The increased susceptibility, in turn, explains the higher rate (>30%) of F1 antibody–positive wild great gerbils during the surveillance of a natural plague focus [9]. In nature, the antibody could persist in great gerbils for more than 200 days (unpublished data), which is similar to the report by Park et al. [30], who showed that the antibody response in great gerbils recovering from a plague infection has seasonal characteristics.

In conclusion, the great gerbil is highly resistant to *Y. pestis* infection, although with great variation between individuals. The persistent *Y. pestis* infection in this animal, which is able to carry the pathogen for a longer time, makes the great gerbil a perfect reservoir for *Y. pestis* in the Junggar Basin, a part of the natural plague focus in Central Asia.
